# Bacteraemia and obstructive pyelonephritis caused by *Bifidobacterium breve* in an elderly woman: a case report and literature review

**DOI:** 10.1099/acmi.0.000574.v3

**Published:** 2023-10-19

**Authors:** Mitsuru Nishio, Hiroshi Morioka, Shun Takai, Yukari Osada, Yoshie Seki, Takato Osugi, Airi Oba, Yuki Miyaki

**Affiliations:** ^1^​ Department of Clinical Laboratory, Komaki City Hospital, Komaki, Aichi, Japan; ^2^​ Infection Control Team, Komaki City Hospital, Komaki, Aichi, Japan; ^3^​ Department of Infectious Diseases, Nagoya University Hospital, Nagoya, Aichi, Japan; ^4^​ Department of Urology, Komaki City Hospital, Komaki, Aichi, Japan; ^5^​ Department of Medical Technique, Nagoya University Hospital, Nagoya, Aichi, Japan

**Keywords:** *Bifidobacterium breve*, urinary tract infection, *Bifidobacterium*, pyelonephritis

## Abstract

*
Bifidobacterium* spp. are non-spore-forming Gram-positive anaerobes that are indigenous to the human gastrointestinal tract and vagina. They are believed to be non-pathogenic organisms for humans and thus are widely used as probiotics. An 83-year-old woman taking cephalexin for 4 days was diagnosed with obstructive pyelonephritis. Y-branched Gram-positive rods were found in both anaerobic and aerobic blood culture bottles, and in an anaerobic urine culture. *Bifidobacterium breve* was finally identified. Ceftriaxone and metronidazole were administered to the patient, and she was discharged after intermittent catheterization for dysuria. Urinary tract infection caused by *Bifidobacterium* spp. is believed to be rare, but it can develop in patients with underlying urological conditions. Recognition of the characteristic morphology and conducting anaerobic urine culture may help in identifying more cases of *Bifidobacterium* urinary tract infections.

## Data Summary

No new data were generated in this case report.

## Introduction

*Bifidobacterium* spp. are non-spore-forming Gram-positive anaerobes that are indigenous to the human gastrointestinal tract and vagina. There are over 50 *Bifidobacterium* species; however, only 11 have been isolated from the human gut and oral cavity [1].

*Bifidobacterium* spp. are functionally very important for intestinal health, and are generally considered to be non-pathogenic [2]. They are widely used as a probiotic and are expected to prevent infections and allergies. However, various infections caused by *Bifidobacterium* spp., (e.g. prosthetic joint infection, pulmonary infection, necrotizing fasciitis and bacteraemia) have been reported [3–6]. Dental caries caused by *Bifidobacterium dentium* is the most common clinical disease involving *Bifidobacterium* spp. [7]. Other species*, Bifidobacterium adolescentis*, *Bifidobacterium breve* and *Bifidobacterium longum,* are occasionally isolated from various infections, primarily in immunocompromised individuals [1, 8–11]. However, urinary tract infections (UTIs) caused by these species have been rarely reported.

## Case presentation

An 83-year-old woman with diabetes mellitus and dementia visited a local clinic complaining of fever. Although the details were unknown, she was prescribed cephalexin.

However, she gradually developed appetite loss and difficulty in ambulation. Four days later, she had persistent fever and appetite loss and could not move. Thus, her family requested an ambulance, and she was transported to our hospital.

The patient’s vital signs were as follows: blood pressure, 156/110 mmHg; heart rate, 140 min^−1^ (sinus tachycardia); respiratory rate, 22 min^−1^ (slight tachypnea); body temperature, 36.4 °C (normal); and Glasgow coma scale score, E4V4M6 (best eye and motor response, but confused verbal response). Neither abdominal tenderness nor costovertebral tenderness was noted in the patient. Other physical examinations were unremarkable. The laboratory findings were as follows: white blood cell count, 6700 µl^-1^ (normal range, neutrophils, 78.6%); creatinine, 6.5 mg dl^−1^ (high); and C-reactive protein, 19.0 mg dl^−1^ (high). The urinalysis results were as follows: pH, 5.0 (low); glucose, 3+; protein 1+; red blood cell count, 11.5/high power field (HPF, high); white blood count, 500/HPF (high); and nitrate, negative (normal). Abdominal CT revealed diffuse bladder wall thickening, bilateral dilation of the renal ureters, hydronephrotic kidneys and perinephric fat stranding; however, there were neither ureteral nor kidney stones.

The patient was diagnosed with obstructive pyelonephritis and admitted to the hospital after foley catheter insertion. Therefore, ceftriaxone 1 g qd was administered intravenously to the patient after obtaining samples for a urinary culture and two sets of blood cultures. The following day, a urologist was consulted. Based on imaging findings and bacteriuria, the urologist determined that she had chronic cystitis. The cause of the obstructive pyelonephritis was unclear; however, invasive procedures were not indicated, considering the patient’s general condition.

**Fig. 1.**
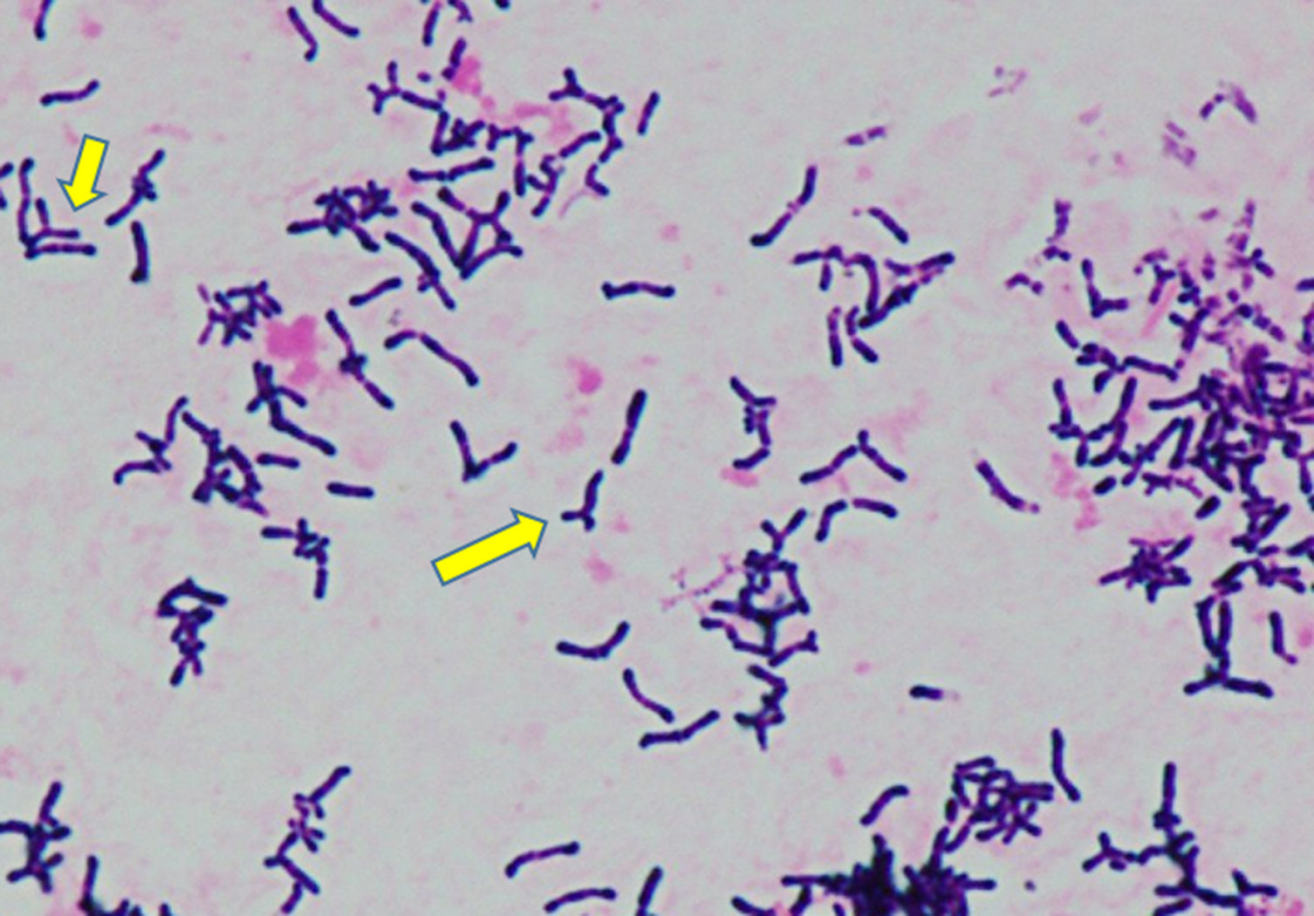
Gram-positive rods at 1,000 x magnification from a blood culture bottle sample (Arrow: Y-branched form).

**Fig. 2.**
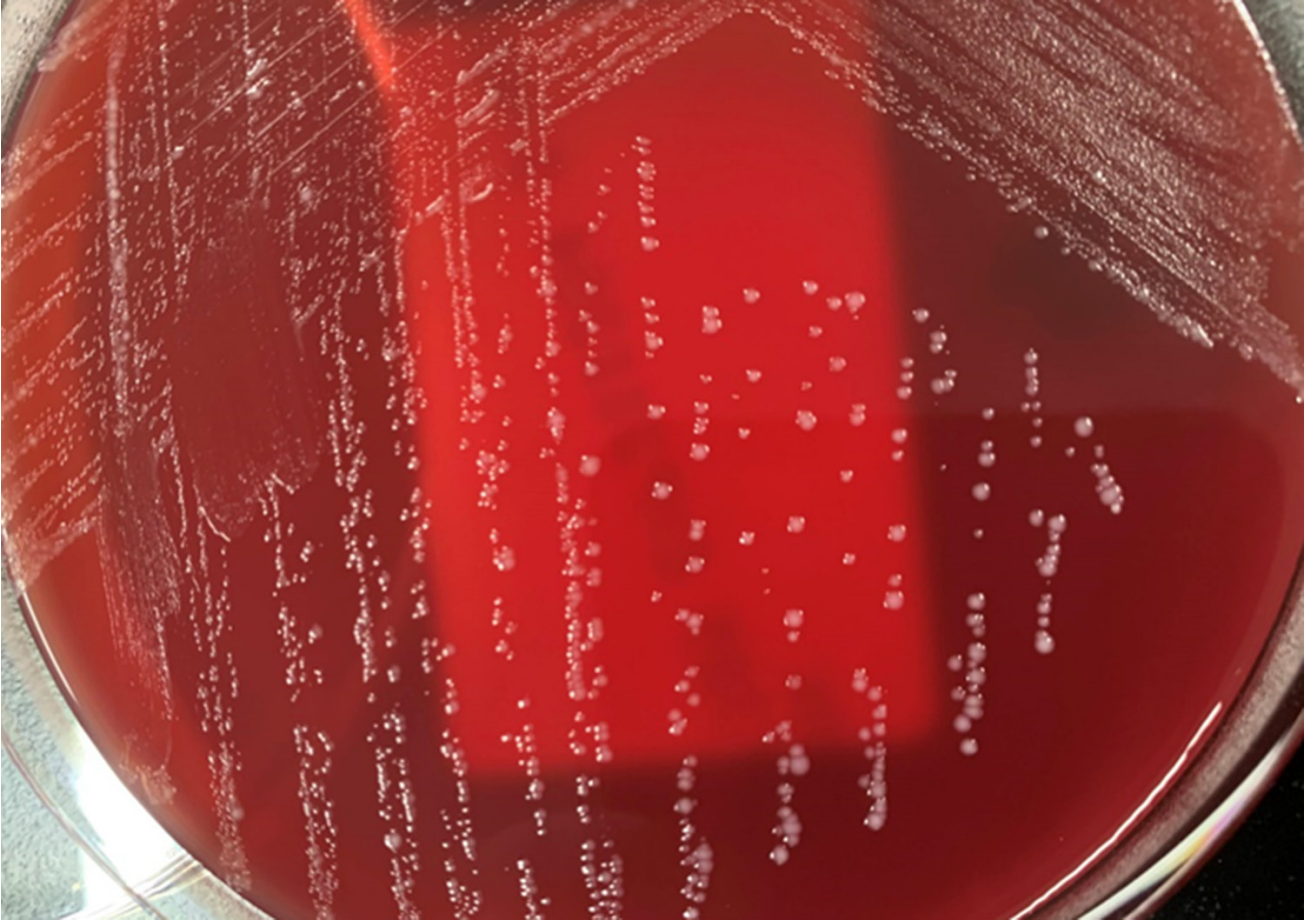
Grey and smooth colonies on the ABHK agar plates after 48 h incubation. na, not applicable; F, female; M, male; nd, no data; MALDI-TOF-MS, matrix-assisted laser desorption/ionization time-of-flight mass spectrometry; UTIs, urinary tract infections; BBG-01, *Bifidobacterium breve* strain Yakult; CTRX, ceftriaxione; ABPC/SBT, ampicillin–sulbactam; CLDM, clindamycin; PCG, penicillin G; CPFX, ciplofloxacin; CTX, cefotaxime; CMZ, cefmetazole; CEZ, cefazolin; VCM, vancomycin; MEPM, meropenem; LZD, linezolid; PIPC/TAZ, piperacillin–tazobactam; MNZ, metronidazole. *Positive bottles are not described in the reference.

Two sets of anaerobic blood culture bottles (BD BACTEC 22F anaerobic medium; Becton Dickinson and Company, Sparks, NV, USA) and one set of aerobic culture bottles (BD BACTEC 23F aerobic medium) gave positive results after 33, 47 and 132 h, respectively. Gram staining from the blood culture bottles revealed Gram-positive rods (GPRs) with Y-branched forms (Fig. 1). The GPRs were incubated at 35 °C in an anaerobic atmosphere on an ABHK agar plate (Nissui Pharmaceuticals, Tokyo, Japan) and in an aerobic atmosphere supplemented with 5 % CO_2_ using BBL trypticase soy agar (TSA) with 5 % sheep blood and chocolate II agar LDIP (Becton Dickinson and Company, Sparks, NV, USA). Grey and smooth colonies were observed on the ABHK agar plates after 48 h (Fig. 2). Colonies smaller than those on ABHK agar were found on BBL TSA agar after a 48 h incubation period. All bacteria developed on ABHK agar plates and TSA with 5 % sheep blood and chocolate II agar were identified by two matrix-assisted laser desorption/ionization time-of-flight mass spectrometry (MALDI-TOF-MS) methods. They were identified as *B. breve* using MBT Compass v. 4.1 and MBT Compass Library v. 9.0.0.0.(8468 MSPs) (Bruker Daltonics, Bremen, Germany; score value 2.11) with a microflex LT/ST system, and as *Bifidobacterium* spp. using VITEK MS software 4.3.0 and VITEK MS Knowledge Base version 3.0 (bioMérieux, Marcy-l’´Etoile, France). The 16s RNA sequences of the amplified products obtained from the organism matched 100 %.

Susceptibility testing was performed using a dry plate EIKEN (Eiken Chemical Co., Ltd, Tokyo, Japan) assay using the microbroth dilution method. Minimum inhibitory concentrations (MICs) were as follows: penicillin G, 0.25 µg ml^−1^; ceftriaxone, 2 µg ml^−1^; cefmetazole, >16 µg ml^−1^; clindamycin, ≦0.25 µg ml^−1^; vancomycin, ≦1 µg ml^−1^; and metronidazole, ≦8 µg ml^−1^.

Gram staining of the urine samples showed only numerous Y-branched GPRs. Antimicrobial-susceptible *Klebsiella pneumoniae* and *Escherichia coli* (MIC: cefazolin ≦1 µg ml^−1^, ceftriaxone ≦0.5 µg ml^−1^, levofloxacin ≦0.12 µg ml^−1^), and *Enterococcus faecalis* (no antimicrobial susceptibility results) grew on BD BBL Prepared Plated: CHROMagar Orientation Media under aerobic conditions, whereas *B. breve* and *Murdociella* sp. (no antimicrobial susceptibility results) grew on the ABHK agar plates under anaerobic conditions.

Following catheter drainage and administration of ceftriaxone, the patient soon became afebrile. The clinical course of this case proceeded well. After the organism was identified as *B. breve *, oral metronidazole 500 mg TID was added to the treatment. Ceftriaxone and metronidazole were administered for 11 and 4 days, respectively. An interview with the patient revealed no history of probiotic or dairy product use. On day 29, the patient was discharged after intermittent catheterization for dysuria.

Two days after discharge from the hospital, the patient was readmitted because of a recurrence of obstructive pyelonephritis, and only extended-spectrum β-lactamase-producing *K. pneumoniae* (MIC: ceftriaxone >1 µg ml^−1^, meropenem≦0.25 µg ml^−1^, levofloxacin 1 µg ml^−1^) were isolated from blood and urine cultures. During the hospital stay, she developed cerebral embolism and died approximately 7 weeks after the *B. breve* bacteraemia episode.

## Discussion

The clinical significance and incidence of infections caused by *Bifidobacterium* spp. are unclear. In Norway, 0–2 bacteraemia cases due to *Bifidobacterium* spp. were reported annually between 2007 and 2012 [6]. Brook *et.al*. reported that 57 *Bifidobacterium* spp. were identified in 2033 samples from paediatric patients. Among these, *Bifidobacterium* spp. could not be identified in 342 blood cultures [8]. Mahlen *et al*. reported three adult cases of *B. breve* bacteraemia during 2000–2007 in two US hospitals [10]. Boume *et al*. reported that 10 *Bifidobacterium* spp. were detected in 91 493 blood cultures from 1972 to 1977 [12]. Probiotics play a major role in *Bifidobacterium* bloodstream infections in children [13, 14]. However, it is unclear whether invasive *Bifidobacterium* infections in adults are associated with probiotics [15].

In total, 14 cases of bloodstream infections with *B. breve* have been reported in the literature (Table 1) [3, 6, 10, 13, 16, 17]. Although *Bifidobacterium* spp. are classified as anaerobes, they can grow in aerobic atmospheres supplemented with 5 % CO_2_. Andriantsoanirina *et al*. reported that many *Bifidobacterium* spp. are resistant to oxygen; in fact, 77.8 % of the evaluated *B. breve* strains were resistant to oxygen [18]. Here, the time to positivity from the blood culture was longer for the aerobic bottles than that for the anaerobic bottles. Moreover, *B. breve* bacteraemia might be underestimated in children taking probiotics because usually only samples for aerobic cultures are collected from children.

**Table 1.** Reviews of bacteremia caused by *Bifidobacterium breve
*

No	Reference	Age/ Gender	Blood culture system	Positive bottle	Polymicrobial bacteremia	Time to positivity (hours)	Identification	Probiotics	Underlying disease	Antibiotics	Duration of antibiotics (days)	Outcome
1	3	42 /M	nd	ND/ anaerobic bottle	–	nd	16S rRNA gene, MALDI-TOF-MS	nd	Necrotizing fasciitisObesity, dyslipidaemia, type 2diabetes mellitus	CTRX, ABPC/ SBT+CLDM	32	Recovered
2	6	49 /F	nd	nd	–	nd	MALDI-TOF-MS	nd	Recurrent wound infection	PCG, CPFX, CLDM	nd	Recovered
3	6	84 /F	nd	nd	Bacteroides *spp*., Candida glabrata	nd		nd	Pyelonephritis, hydronephrosis caused by a kidney stone	CTX	nd	Recovered
4	10	ND/ND	BacT/ Alert 3D^TM^	BacT/ Alert FN or SA*	nd	nd	16S rRNA gene	nd	History of decubitis ulcers, frequent admissions for recurrent UTIs	nd	nd	nd
5	10	ND/ND			–	nd		nd	Peritonitis	nd	nd	nd
6	10	ND/ND			Bacteroides vulgatus Fusobacterium *spp*.	nd		nd	Stage B prostate cancer, ileal resection; thought to be transient bacteremia	nd	nd	nd
7	13	0 /M	BACTEC 9050 ^TM^	94F paediatric bottle	–	170	polymorphic DNA analysis	BBG-01	Cloacal exstrophy, postoperative adhesive ileus	CMZ, CEZ, VCM	15	Recovered
8	13	0 /F			–	138			Esophageal atresia, postoperative gastro-esophageal reflux, aspiration pneumonia	CEZ, VCM	14	Recovered
9	13	0 /M			–	128			Necrotizing enterocolitis, gastrointestinal perforation	ABPC/ SBT, MEPM, VCM, LZD	14	Recovered
10	13	0 /M			–	114			Necrotizing enterocolitis, gastrointestinal perforation, congenital heart diseases	PIPC/ TAZ, VCM	20	Recovered
11	13	0 /M			–	125			Food protein-induced enterocolitis syndrome	ABPC/ SBT, VCM	14	Recovered
12	13	0 /F			–	229			Ileal volvulus, food protein-induced enterocolitis syndrome	CMZ	5	Recovered
13	16	0/ND	nd	nd	–	4 days	polymorphic DNA analysis	BBG-01	Omphalocele	ABPC/ SBT, MEPM	12	Recovered
14	17	2 /M	nd	ND/anaerobic bottle	–	nd	MALDI-TOF-MS	–	Leukaemia, febrile neutropenia	PIPC/ TAZ, PCG	14	Recovered
This case	na	83 /F	BACTEC FXrt 3D^TM^	22F anaerobic medium	–	33, 47	16S rRNA gene, MALDI-TOF-MS	–	Obstractive pyelonephritis, diabetes mellitus	CTRX+MNZ	11	Recovered
				23F aerobic medium		132						

*Positive bottles are not described in the reference.

ABPC, ampicillin; ABPC/SBT, ampicillin sulbactam; AIHA, autoimmune hemolytic anaemia; ; BCX, blood culture; CP, chloramphenicol; CTFX, ceftriaxione; CTX, cefotaxime; DM, diabetes mellitus; ESBL, extended-spectrum β-lactamase ; F, female; LT, Left; M, male; MALDI-TOF-MS, matrix-assisted laser desorption/ionization time-of-flight mass spectrometry; MCFG, micafungin; MDS, myelodysplastic syndromes; MEPM, meropenem; MNZ, metronidazole; NA, not applicable; ND, no data; SSPE, subacute sclerosing panencephalitis; UTI, urinary tract infection.

To our knowledge, UTIs caused by *Bifidobacterium* spp. are quite rare; we only found nine cases (Table 2) [5, 10, 19–23]. Most of the patients had underlying diseases and urological problems. We suspected the following aetiology for the present case: chronic cystitis led to obstructive pyelonephritis caused by polymicrobial pathogens; preceding cephalexin selected *B. breve *, which is naturally resistant to cephalosporins; and, *B. breve* bacteraemia developed due to increased pressure in the renal pelvis. Here, Gram staining of urine specimen showed numerous typical Y-branched GPRs; however, no growth was observed in the routine urine culture. Anaerobic urine culture should be considered in patients with an immunocompromised state, urological problems and positive Gram staining but negative routine culture [24]. If *Bifidobacterium* spp. are suspected based on Gram staining and poor growth of routine urine culture, anaerobic culture is the key to the early identification of this organism.

**Table 2.** Reviews of UTIs caused by *Bifidobacterium* spp

No.	Reference	Age/gender	Underlying diseases	Urological problems	History of UTI	Bcx	Probiotics	Urine culture	Identification	Antibiotics	Duration (days)	Outcome
1	5	51/F	DM, hypertension, cervical carcinoma	Obstructive nephropathy	nd	nd	nd	* Bifidobacterium* spp. *E. coli* (ESBL+)	MALDI-TOF-MS, VITEK^®^ 2	MEPM	nd	Recovered
2	10	nd/nd	Dementia	nd	nd	nd	*nd*	* B. scardovii *	16S rRNA gene	nd	nd	nd
3	10	nd/nd	nd	nd	nd	nd	nd	* B. breve *	16S rRNA gene	nd	nd	nd
4	19	66/F	MDS, liver cirrhosis, DM	Lt hydronephrosis, Ureter stone	+	nd	*nd*	* Bifidobacterium* spp. *C. glabrata*	RapID ANA II System	MEPM	7	Recovered
5	20	80/F	Breast cancer, hypothyroidism, AIHA	nd	+	nd	nd	* B. scardovii *	16S rRNA gene	ABPC/SBT	5	Relapsed
6	21	40s/M	SSPE, DM	Bilateral hydronephrosis	–	–	–	* B. breve *	16S rRNA gene	ABPC/SBT	nd	Recovered
7	22	7/F	nd	Recurrent UTIs	+	nd	nd	*B. adolescentis, P. asaccharolyticus*	Conventional methods	ABPC	10–14	Recovered
8	23	41/M	Abdominal injury	Urethral stricture and repeated urethral dilation	+	+	–	* B. adolescentis *	nd	CP	10	Recovered
This case	na	83/F	DM, hypertension	Bilateral hydronephrosis	+	+	–	*B. breve, K. pneumoniae, E. coli, E. faecalis*	16S rRNA gene, MALDI-TOF-MS	CTRX, MNZ	11	Recovered

ABPC, ampicillin; ABPC/SBT, ampicillin sulbactam; AIHA, autoimmune hemolytic anemia; Bcx, blood culture; CP, chloramphenicol; CTRX, ceftriaxione; CTX, cefotaxime; DM, diabetes mellitus; ESBL, extended-spectrum β-lactamase; F, female; Lt, left; M, male; MALDI-TOF-MS, matrix-assisted laser desorption/ionization time-of-flight mass spectrometry; MCFG, micafungin; MDS, myelodysplastic syndromes; MEPM, meropenem; MNZ, metronidazole; NA, not applicable; ND, no data; SSPE, subacute sclerosing panencephalitis; UTI, urinary tract infection.

Sequencing of the 16s RNA gene has been used as the gold standard for accurate species identification [1, 25]. However, genetic analysis is difficult to perform in actual clinical practice. The identification of *Bifidobacterium* spp. based on phenotypic characteristics is challenging [26]. Identification by biochemical testing is known to be affected by insufficient growth and poor reproducibility [1]. According to the manufacturer’s instructions, the three commercial kits that can identify *Bifidobacterium* spp. in each database based on biochemical characterization are the BD BBLTM CRYSTALTM ANR ID System (Becton Dickinson and Company, Sparks, NV, USA), which can identify *B. adolescentis *, *B. dentium* and *Bifidobacterium* spp.; the API 20A (bioMérieux, Marcy-l’Etoile, France), which can identify *B. adolescentis *, *B. dentium, B. breve, B. bifidum* and *Bifidobacterium* spp.; and the API RAPID ID 32A (bioMérieux, Marcy-l’Etoile, France), which can identify *B. adolescentis *, *B. breve *, *B. longum *, *B. dentium, B. bifidum* and *Bifidobacterium* spp. [27, 28]. If microbiology technicians think that anaerobic bacteria are absent due to their growth in aerobic bottles, an incorrect identification kit may be used, which can result in misidentification of the bacteria. Although identifying the bacteria to species level by biochemical characterization is sometimes difficult, identification to the genus level is possible if the correct identification kit is selected. Characteristic Gram staining will help in identifying the *Bifidobacterium* spp. without MALDI-TOF-MS or sequencing of the 16s RNA gene.

MALDI-TOF-MS has been reported to be useful in identifying anaerobic bacteria, including *Bifidobacterium* spp. [29, 30]. The performance of MALDI-TOF-MS has been believed to surpass that of these commercial kits, with a higher rate of correct identification and fewer misidentifications. As such, the implementation of MALDI-TOF-MS has become a cornerstone in the identification of anaerobic bacteria, including our hospital [1, 31]. Weber *et al*. reported an adult case of *B. longum* bacteraemia identified using MALDI-TOF-MS (Bruker Daltonics) [15]. Esaiassen *et al*. reported that MALDI-TOF-MS (Microflex LT instrument, Bruker Daltonics) can be used for species-level identification of *B. longum *, *B. breve* and *B. animalis* [6]. In our case, Vitek MS did not provide a species-level identification. *B. breve* is included in *Bifidobacterium* spp. in VITEK MS Knowledge Base version 3.0–3.2, thus identification of subspecies in this case was impossible using VITEK MS. The accurate identification and compilation of clinical pictures by expanding the library of MALDI-TOF-MS or identification kits will help in identifying differences in the clinical pictures of different *Bifidobacterium* spp. infections. A greater compilation of *Bifidobacterium* infection reports will lead to clarifying the pathogenicity, clinical picture and optimal management of infections, especially UTIs.

## Conclusion

In this report, we describe a rare case of *B. breve* bacteraemia and obstructive pyelonephritis. The combination of *Bifidobacterium* bacteraemia and UTI is believed to be rare; however, there may be undiagnosed cases due to the poor growth in routine urine culture and the difficulty of identification. Recognition of Y-branched GPR and conducting anaerobic urine culture may lead to find more cases of *Bifidobacterium* UTIs. Clinicians and microbiology technicians need to keep in mind the usefulness and limitation of commercial kits and MALDI-TOF-MS in identifying *Bifidobacterium* spp.
